# An Innovative Enzymatic Surface Plasmon Resonance-Based Biosensor Designed for Precise Detection of Glycine Amino Acid

**DOI:** 10.3390/bios15020081

**Published:** 2025-02-01

**Authors:** Gabriela Elizabeth Quintanilla-Villanueva, Osvaldo Rodríguez-Quiroz, Araceli Sánchez-Álvarez, José Manuel Rodríguez-Delgado, Juan Francisco Villarreal-Chiu, Donato Luna-Moreno, Melissa Marlene Rodríguez-Delgado

**Affiliations:** 1 Centro de Investigaciones en Óptica AC, Div. de Fotónica, Loma del Bosque 115, Col. Lomas del Campestre, León 37150, Guanajuato, Mexico; quintanillagabriela@cio.mx (G.E.Q.-V.); osvaldo.rodriguez@cio.mx (O.R.-Q.); dluna@cio.mx (D.L.-M.); 2 Universidad Tecnológica de León, Electromecánica Industrial, Blvd. Universidad Tecnológica #225, Col. San Carlos, León 37670, Guanajuato, Mexico; asalvarez@utleon.edu.mx; 3 Tecnológico de Monterrey, School of Engineering and Sciences, Av. Eugenio Garza Sada Sur 2501, Col. Tecnológico, Monterrey 64849, Nuevo León, Mexico; jmrd@tec.mx; 4 Universidad Autónoma de Nuevo León, Facultad de Ciencias Químicas, Av. Universidad S/N Ciudad Universitaria, San Nicolás de los Garza 66455, Nuevo León, Mexico; 5 Centro de Investigación en Biotecnología y Nanotecnología (CIByN), Facultad de Ciencias Químicas, Universidad Autónoma de Nuevo León, Parque de Investigación e Innovación Tecnológica, Km. 10 autopista al Aeropuerto Internacional Mariano Escobedo, Apodaca 66629, Nuevo León, Mexico

**Keywords:** surface plasmon resonance, glycine, laccases, enzymatic biosensor

## Abstract

Glycine is an essential amino acid involved in synthesizing a variety of important biomolecules, and its concentration can influence numerous biochemical processes, including the severity of symptoms in a wide range of conditions in humans, such as cancer, schizophrenia, major depression, and diabetes. While a few costly or labour-intensive methods are currently available, we have developed a new enzymatic biosensor that can accurately measure glycine levels with remarkable simplicity. By employing immobilized laccase enzymes in combination with a surface plasmon resonance (SPR) device, our system achieved a limit of detection (LOD) of 9.95 mM and a limit of quantification (LOQ) of 33.19 mM. In addition, it demonstrated a recovery rate of 97.64 ± 7.71%. Moreover, the biosensor maintained consistent signal intensity over 21 days and supported a total of 60 analyses using the same immobilized enzyme setup, demonstrating excellent reusability. Notably, this study marks the first time glycine has been determined using an enzymatic SPR-based platform.

## 1. Introduction

Glycine, the simplest of the amino acids, is a crucial precursor for several important low-molecular-weight metabolites, including purines, porphyrins, glutathione, creatine, and uric acid [1]. In addition to its role in core metabolic pathways, anti-oxidative reactions, and neurological function [2,3], glycine is also an essential component of structural proteins such as collagen and elastin [4]. These proteins play a significant role in the composition of connective tissues and extracellular matrices [5]. Moreover, glycine is an important component of bile acids, which help promote the absorption of fats and fat-soluble vitamins. It can also act as a signalling molecule in both metabolic and non-metabolic pathways [6].

Glycine, although synthesized in the body from serine, choline, sarcosine, and glyoxylate through metabolic processes in the liver and kidneys, is often regarded as a conditionally essential amino acid. This classification arises because the body’s natural production is usually insufficient to meet its metabolic needs [7,8]. As a result, dietary glycine supplementation may be necessary to promote a healthy lifespan [9,10]. Low plasma concentrations of glycine have been associated with several metabolic disorders, including obesity, type 2 diabetes, and non-alcoholic fatty liver disease, particularly when compared to healthy individuals [11,12]. Such deficiencies have also been noted with schizophrenia [13]. While these conditions may not be immediately life-threatening, a lack of available exogenous glycine can contribute to tumour formation due to cellular stress response [14]. Glycine deficiency has been associated with malignant transformations, such as pancreatic cancer [15], and in the NCI-60 cell line panel, where 60 different cancer cell lines were tested [16]. Conversely, elevated levels of glycine in blood serum and saliva have been correlated with various types of cancer, including colorectal [17], lung, bladder, prostate, kidney, and colon cancers [15]. These high levels may also play a significant role in brain metastasis [18]. Therefore, monitoring glycine levels could be essential for diagnosing specific health conditions, guiding appropriate treatments, and assessing the nutritional content of food products and dietary supplements.

Accurate monitoring of glycine concentrations is valuable across various sectors, including nutritional sciences, biotechnology, environmental analysis, and, notably, the assessment of glycine levels in blood serum. Unfortunately, there have been a limited number of studies focused on clinical samples. For instance, Jiang and collaborators [19] developed a method for determining glycine in cerebrospinal fluid by ion-pair reversed-phase liquid chromatography–tandem mass spectrometry (LC-MS/MS). Their method detected a mean endogenous glycine concentration of 748 ± 30.6 ng/mL. In a similar study, Andreadou et al. [20] detected glycine in blood plasma with average concentrations of 205.3 µM by high-performance liquid chromatography (HPLC) in individuals with amyotrophic lateral sclerosis. In another study, Luu et al. [15] analysed glycine, serine, and their related metabolites in blood serum using gas chromatography–tandem mass spectrometry (GS-MS/MS), finding glycine concentrations ranging from 356.96 to 369.24 µM in healthy people, compared to 337.18 to 354.00 µM in pancreatic cancer cases. While these established methodologies for glycine quantification are powerful and precise, they also have inherent drawbacks. These methods often require expensive equipment and involve labour-intensive sample pretreatment and analysis protocols. Consequently, there has been a push for novel alternatives that are simpler and more cost-effective. This demand for faster more reliable analytical methods for glycine monitoring is especially pertinent in clinical analysis.

In this context, immobilized enzymes have become widely used for the detection of medically important molecules [21]. When integrated with devices that utilize surface plasmon resonance (SPR), these enzymes can offer high accuracy along with a very low limit of detection (LOD) and limit of quantification (LOQ). For example, in a study by Jabbari et al. [22], laccase enzymes from *Bacillus* sp. HR03 were employed to detect bromocriptine, an ergoline derivative critical for treating Parkinson’s disease, achieving a LOD of 0.001 ng mL^−1^. Similarly, Miyazaki et al. [23] utilized urate oxidase to detect uric acid, reaching an LOD of 0.27 μmol L^−1^. Also, Zhang et al. [24] used glucose oxidase as a bioreceptor to detect glucose in urine, achieving a sensitivity of 3.10 pm (mg dL^−1^).

Using enzymes as bioreceptors in SPR devices presents several significant advantages. For instance, sensors equipped with enzymatic reaction devices can be reused repeatedly without losing stability. This not only reduces waste but also lowers costs [25]. The operation of the SPR device is straightforward and rapid, ensuring that the operator is not exposed to hazardous samples. Consequently, this method does not require specialized skills to operate [24]. Samples can be loaded directly into the device without extensive preparation steps [26], and real-time measurements can be conducted [27,28]. In addition to its high sensitivity [29], the platform can be easily modified to detect different enzymatic reactions by simply changing the enzyme or substrate used. For example, laccases, which interact with molecules that contain glycine, have been employed to detect glyphosate [30,31,32]. This study proposes the use of laccase-based receptors integrated with SPR technology for the rapid detection of glycine. This versatile approach allows for real-time measurements and can be applied across various fields, including biomedical diagnostics and the assessment of nutritional content in food products or dietary supplements.

## 2. Methodology

### 2.1. Materials and Reagents

**Figure 1:**
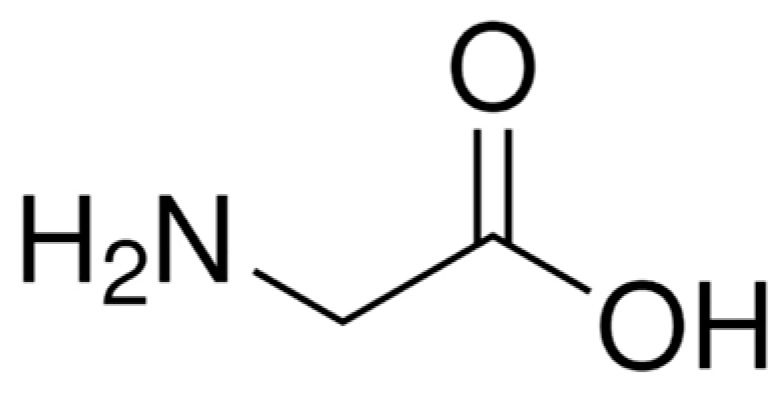
Chemical structure of glycine.

The chemical reagents used for immobilizing the bioreceptor included 16-mercaptohexadecanoic acid (MHDA), 11-mercaptoundecanol (MUD), 1-ethyl-3-(3-dimethylamino-propyl) carbodiimide hydrochloride (EDC) ethanolamine hydrochloride, N-hydroxysuccinimide (NHS), and laccase enzymes from *Rhus vernicifera*. For the study, the Glycine ReagentPlus^®^ pharma grade standard (≥99% by HPLC, code G7126) was employed, which meets the analytical specification of the European Pharmacopoeia (Ph. Eur.) and the United States Pharmacopeia (USP), with a purity range of 99–101%. The chemical structure of the molecule is illustrated in Figure 1. All reagents were of reagent grade and purchased from Sigma-Aldrich (St. Louis, MO, USA). Blood samples were collected through venous puncture from one individual, and the serum was harvested after centrifugation.

The gold chips of the SPR system consisted of thin films of Cr/Au deposited on thin glass substrates of 1 × 1 cm. The process involved the thermal evaporation of a 3 nm chromium layer followed by a 50 nm gold film deposited at a rate of 5 Å/s and 8 × 10^−6^ mbar employing a quartz crystal microbalance thickness monitor (Leybold Inficon XTC/2 Depositions Controllers).

The statistical analysis was performed using Minitab^TM^ Statistical Software (https://www.minitab.com/en-us/products/minitab/, accessed on 11 December 2024; Minitab Inc., State College, PA, USA) and OriginPro 2020b software (https://www.originlab.com/2020b, accessed on 11 December 2024; OriginLab Corporation, Northampton, MA, USA).

### 2.2. SPR Platform

**Figure 2:**
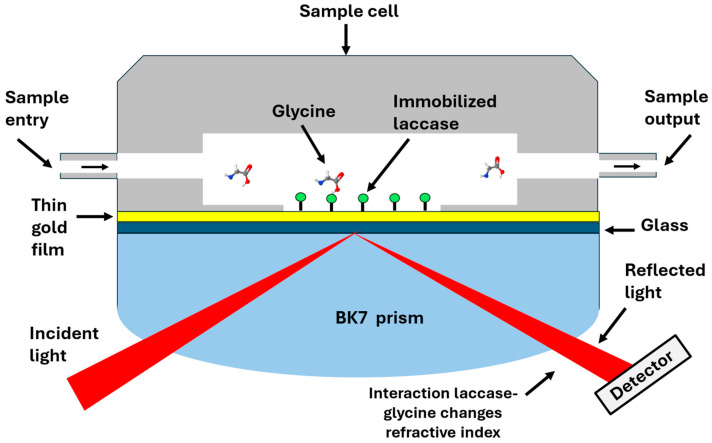
Schematic representation of the biosensing process for glycine detection. The sample is injected into the sample cell, and the analyte (glycine) interacts with the laccases immobilized on the thin gold film, provoking changes in the refractive index and perceiving by the detector as variations in the light intensity.

The SPR setup used in this study was previously described by Sánchez-Alvarez et al. [33] and is based on a Kretschmann configuration. The platform consists of two stacked rotation plates synchronized to move in a θ-2θ system using a stepper motor. Samples were injected into a Teflon cell by a syringe pump (Legato 100) and were faced against a thin gold chip optically coupled to a hemicylindrical BK7 glass prism with an oil-matching index (n = 1.51). Detection is performed using a photodetector (Hamamatsu, model S1226-8Bk) that captures the reflection changes of the He-Ne laser light (Uniphase mod. 1101P) passing through the prism. The plasmonic sensor is based on the optical phenomenon of surface plasmon waves, which are produced when a polarised light incident in the interface of the glass prism (dielectric) and a gold film (metallic layer), waving the electrons of the metal that form an evanescent field propagating along the gold film system [34]. Then, the gold’s surface becomes sensitive to the binding of biomolecules on it, which is reflected as a change in the surface’s refractive index; hence, the reflected intensity is measured by the equipment [34]. Figure 2 provides a schematic representation of the SPR biosensor.

### 2.3. Immobilization of the Enzyme by Covalent Binding

The immobilization process involved a covalent reaction between the enzymes and the gold chips. First, the chips were treated by immersion in acetone and ethanol, then air-dried and incubated for 12 h at room temperature in a solution of MHDA:MUD alkanethiols in ethanol at a final concentration of 250 μM. The free carboxyl groups generated from the alkanethiols are activated with a solution of the EDC/NHS cross-linkers (0.2 M/0.05 M) in MES buffer (100 mM, 500 mM NaCl, pH 5). After this activation step, 200 U mg^−1^ of the enzyme was added to the chip’s surface, allowing the formation of amide bonds between the amino acids of the enzymes and the terminal carboxyl group of the alkanethiols. To prevent nonspecific binding in further analyses, an ethanolamine solution (1 M, pH 8.5) was added to bind the remaining available carboxyl groups after the enzymes were immobilized. The laccase enzymatic activity was determined before and after immobilization by the spectrophotometric UV-Vis assay previously reported [25]. In this assay, 200 µL of the enzyme was added to a reaction solution comprised of 10 mM of ABTS (2,2′-casino-bis (3-ethylbenzothiazoline-6-sulfonic acid)) as substrate in 0.1 M sodium acetate buffer, pH 4.5. The enzymatic reaction changes the absorbance solution, recorded at 420 nm in a UV-Vis spectrophotometer (Cary 50, Varian). The activity unit (U) was expressed as the amount of enzyme necessary to produce 1 µM of product per minute.

### 2.4. SPR Measurements for Glycine Detection

To test the SPR system, all tests were conducted at pH 7.4 to replicate the typical conditions of human blood serum [35], which would be the matrix of interest during this study. For the SPR system assay, the reaction mixture was injected at a flow of 0.2 mL min^−1^ to interact with the immobilized enzymes (bioreceptor). The reaction solution consisted of 400 µL of buffer Tris-HCl (pH 7.4, 250 mM), 1000 µL of 500 mM Glycine (Gly), and 600 µL double-distilled water. The enzymatic activity of laccase at various Gly concentrations enabled the calculation of the analytical parameters of the SPR system. A Gly calibration curve, ranging from 0 to 500 mM, was prepared in 50 mM Tris–HCl buffer (pH 7.4). In this case, the tested concentrations were in the order of mM units, commonly reported as unhealthy physiological levels in glycine-related diseases [36], since laccase presented a diminished activity at lower concentrations (μM). Finally, the regeneration of the bioreceptor (laccase) was accomplished with a 40 mM NaOH solution, injected between each sample analysis, while double-distilled water was used as a blank solution.

The analytical parameters reported for this method included the correlation coefficient (R), limit of detection (LOD), and limit of quantification (LOQ), which were calculated from the calibration curve. The LOD and the LOQ were calculated according to Equations (1) and (2), where *SDb* is the standard deviation of the blank solution and *m* corresponds to the slope of the calibration curve.

LOD = (3 ∗ *SDb*)/*m* (1)

LOQ = (10 ∗ *SDb*)/*m* (2)

Finally, duplicate analysis of blood serum samples spiked with 130 mM Gly was conducted to calculate the percent recovery rate and validate the method in a real matrix. The blood serum was obtained by allowing the blood to clot undisturbed at room temperature for 30 min. The clot was removed by centrifuging at 2000× *g* for 10 min, and the supernatant was stored at −18 °C until use. The experimental recovery obtained in the assay was compared with the sample’s theoretical concentration using a T-test to determine the statistical significance of the results. In terms of reusability of the system, several stock solutions of 500 mM Gly were continuously analysed to observe if the signal decreased over time using the same gold chip with immobilized enzymes.

## 3. Results and Discussion

### 3.1. Immobilization of the Enzyme by Covalent Binding

**Figure 3:**
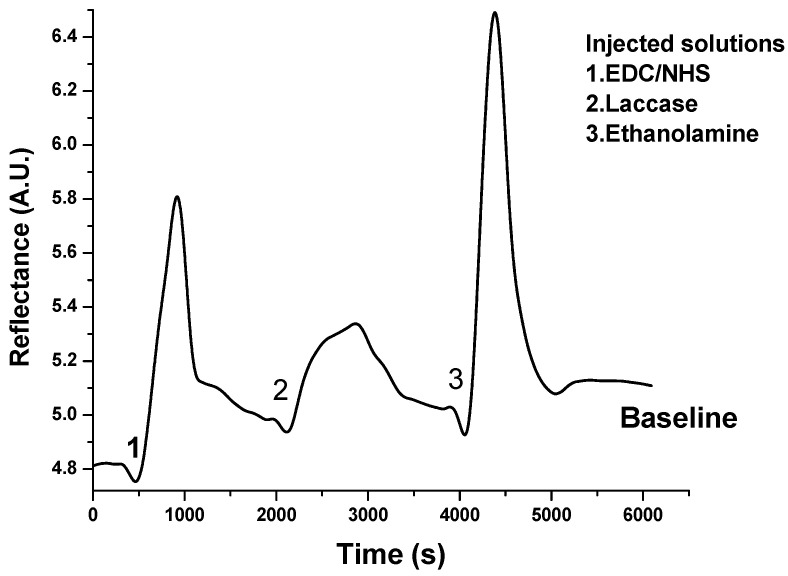
Real-time SPR sensogram showing each step of the laccase immobilization. The process involves the (1) injection of EDC/NHS cross-linkers to activate the surface and (2) the subsequent addition of the enzyme for their attachment through amide bonds. The immobilization is finalized by (3) injecting an ethanolamine solution, which is added as a blocking agent.

The construction of the Gly-sensitive chips was carefully monitored at each stage by tracking the intensity of the reflectance signal in real time. A distinct peak was observed when the reactant molecules passed over the gold chip and adhered to the surface of each layer. After completing each layer, a washing step was performed to remove weakly bonded molecules. This process resulted in a chip with a final enzymatic activity of 80 U mg^−1^ of immobilized laccase. The sensogram obtained from the in-flow immobilization process is shown in Figure 3, illustrating the baseline increase due to the successful linkage of the enzymes.

### 3.2. SPR Measurements for Glycine Detection

**Figure 4:**
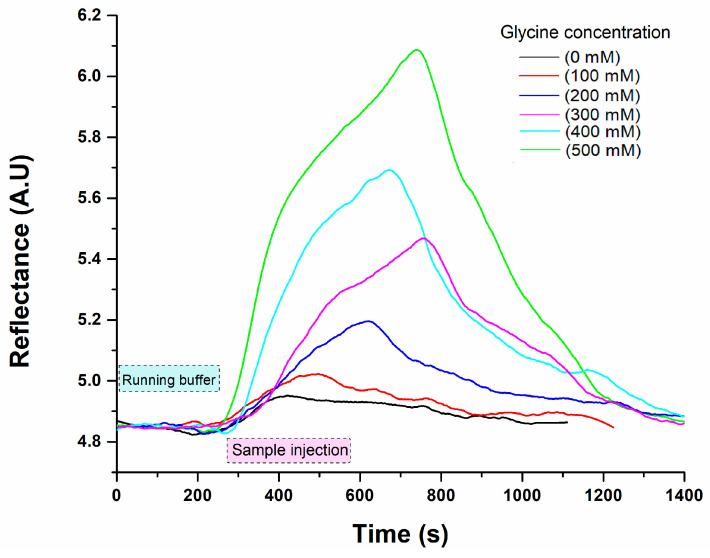
Real-time SPR sensograms of glycine detection in buffer Tris-HCl (50 mM, pH 7.4). Each plotting represents the mean of three replicates (n = 3) of glycine solution at different concentrations.

The detection of glycine was carried out using a direct enzyme-substrate assay on an SPR chip immobilized with laccase enzymes. The oxidation of glycine, catalysed by the laccase enzymes, was monitored using the SPR system. The changes in the refractive index were attributed to the shift in the enzyme conformation due to the binding of the analyte to the enzyme’s active site [37]. The catalytic reaction occurs when the substrate (glycine) interacts with the enzyme (laccase) active site; such enzyme–substrate interactions induce structural changes in the enzyme that increase its hydrodynamic radius, causing a net increment in refractive index on the surface (SPR signal) [37]. The increase in linearity in the refractive index is mainly attributed to the number of molecule interactions bound to the sensor surface. Similarly, Jabbari et al. [22] demonstrated the detection of electron transfer in the oxidation process catalysed by laccase using the SPR method. Another study evaluated the detection of bromocriptine using the SPR technique coupled with laccase from *Bacillus* sp. HR03. During the assay, the reaction mixture was injected at a flow of 0.2 mL min^−1^, and the enzymatic activity of laccase towards different glycine concentrations enabled the determination of the analytical parameters of the SPR system. The sensogram results from the Gly solutions, ranging from 0 to 500 mM, can be observed in Figure 4. The sample solutions were prepared in 50 mM Tris–HCl buffer (pH 7.4), and a regeneration solution of 40 mM NaOH was injected between each sample analysis. The reflectance intensity signal related to the glycine content during the binding event with the laccase is observed through time, showing a higher signal as the concentration increases.

**Figure 5:**
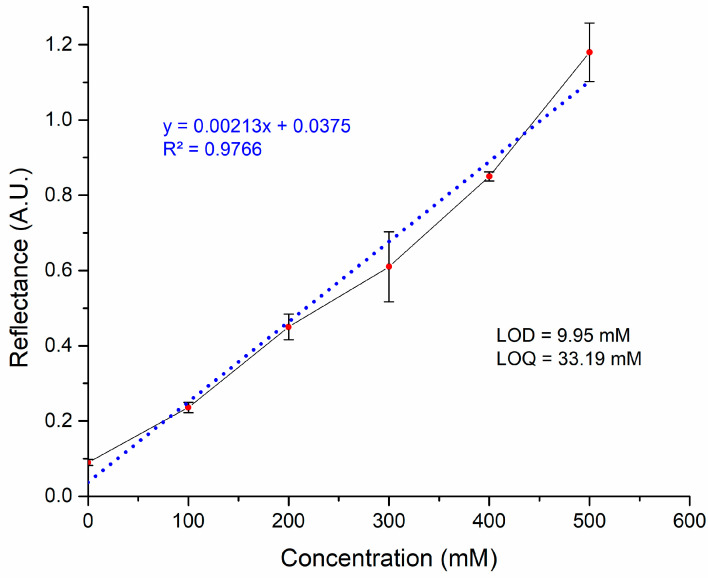
Calibration curve of the maximum reflectance intensity of glycine solutions in PBS buffer at different concentrations. The experimental data (●) reflect the ± standard deviation of the measurements (n = 5) and the linearity fitting (blue dotted line).

The signals obtained from measuring each glycine solution, as shown in the sensograms, were used to create a calibration curve based on their concentrations. The results of this curve, presented in Figure 5, demonstrated a strong linearity (R^2^ = 0.9766) with a working range of 0 to 500 mM. Additionally, the LOD was found to be 9.95 mM, while the LOQ was 33.19 mM. Unfortunately, the laccase enzyme presented low activity at concentrations of glycine in the order of μM, considered as healthy levels in biological fluids (commonly from 25 to 500 μM), showing detection solely at high concentrations in mM, which are reported as unhealthy levels in glycine-related diseases [36]. For example, Luu et al. stated that typical Gly concentration in plasma ranges from 356.9 to 369.2 µM [15]. Although our biosensor may not be suitable for medical use at healthy concentrations, it could act as an alarm in cases where the glycine reaches levels that lie outside of the average, with the advantage that no sample dilution would be necessary to be precise during the clinical analysis.

**Figure 6:**
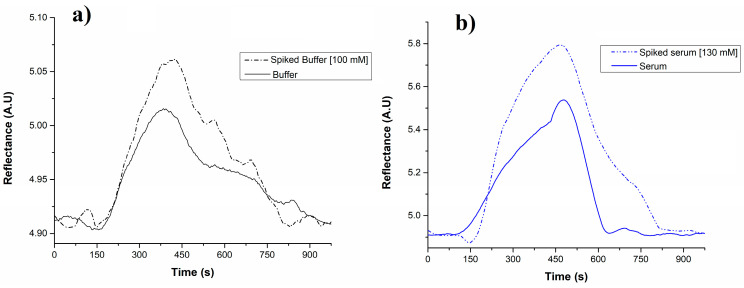
Evaluation of the level of nonspecific signals (matrix effects) in (**a**) PBS buffer and (**b**) serum samples. Negative controls included unspiked PBS buffer (blank solution) and serum (without glycine), and serum samples (with glycine at 100 and 130 mM, respectively) were used as positive controls (n = 3).

It is important to recognize that certain components present in real samples may cause interferences, leading to false positives during analysis [38]. Therefore, assessing the method’s performance under actual analysis conditions is essential. In this study, PBS buffer and serum samples were used as controls to ensure that the sample matrix did not produce false positives or false negatives (see Figure 6). The negative controls consisted of unspiked PBS buffer (blank solution) and serum (without glycine), while spiked PBS buffer and serum samples (with glycine at 100 and 130 mM, respectively) served as positive controls. In the negative control buffer solution, a background signal was observed that exceeded 10% compared to the baseline. Similarly, the negative control serum sample displayed a matrix effect, showing a 14% increase over the background signal observed in the buffer solution. However, we cannot conclusively attribute these background signals solely to matrix effects (interferences in the solutions) because a similar effect was noted in the phosphate buffer signal background, where no interferences are expected (as it is composed only of phosphate salts and water). Therefore, it is likely possible that the instrumental noise from the developed biosensor is also contributing to the background signal. Blood serum samples were spiked with 130 mM of glycine and analysed in triplicate to determine the percent recovery rate. Despite the potential matrix effect observed, the resulting recovery percentage was 97.64 ± 7.71%. We compared the experimental recovery from the assay with the theoretical concentration of the sample using a T-test to assess the statistical significance of the results. The T-test is a statistical method used to determine if there is a significant difference between the means of two groups. With *p* = 0.05, n = 3, t calculated = 6.94, and t critical = 12.706, the results showed no significant difference, indicating that the biosensor’s measurements were consistent with the expected values. Nonetheless, this method should be regarded as an initial stage toward further investigations in optimizing and validating the optical analysis. It is essential to evaluate non-specific interactions with unrelated compounds, such as ascorbic acid, glucose, lactate, or other amino acids present in real samples, to accurately identify possible matrix effects. In terms of the enhancement of laccase performance, exploring novel redox mediators or genetic modifications could be interesting to test.

**Figure 7:**
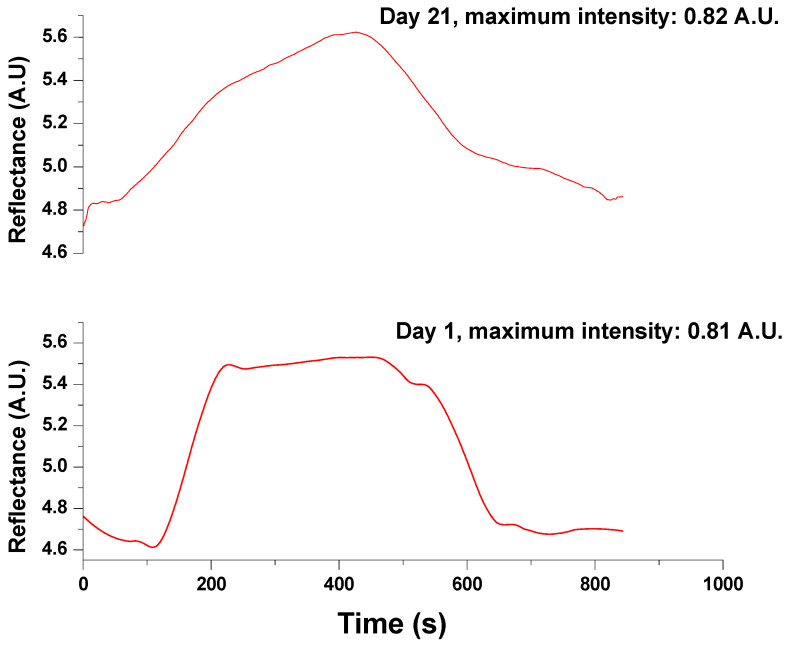
Evaluation of biosensor reusability by comparison of the reflectance intensity. The test was performed with a stock of glycine 500 mM in buffer Tris-HCl (pH 7.4) on day 1 and day 21 (n = 2).

On the other hand, a reusability test was conducted on the system. Stock solutions of 500 mM glycine were continuously injected to observe whether the signal decreased over time when using the same gold chip with immobilized enzymes (see Figure 7). The immobilized system endured 60 regeneration cycles using 40 mM NaOH as a regeneration solution before any significant loss of recognition capacity. The biosensor on day 21 required almost 400 s to reach the highest reflectance but only showed a slight variation of 1.22% over the 21 days. A T-test indicated that there was no significant difference (*p* = 0.05 and *n* = 2). This variation may be attributed to enzyme damage, which could lead to a less stable enzyme–substrate reaction resulting from the continuous flow of the samples and the regeneration steps involving the addition of NaOH.

Although the enzymes could be damaged after many cycles of use, immobilization is a good strategy to keep the enzymes active for longer. For example, evidence suggests that immobilization contributes to the long-term stability of laccases. For example, Ranimol and Sunkar [39] conducted a comparative study on the enzymatic activity of free and immobilized laccase. Their findings revealed that the free enzyme retained 32.44% of its initial activity, while the immobilized system retained 70.21% [39]. In another experiment, Ashrafi et al. [40] reported that laccase maintained 63% of its activity after 16 days. Additionally, Rouhani et al. [41] observed that immobilized laccase retained 88% of its initial activity after storage for 20 days. The reusability of enzymes is crucial for cost-effectiveness and overall efficiency improvement [42]. Consequently, our biosensor’s stability enhances sustainability by reducing the quantity of required enzymes and reagents, such as cross-linkers, lowering costs.

Table 1 compares some analytical parameters for methods reported in glycine detection; unfortunately, most of the approaches lack appropriate detail of analytical data, such as selectivity, linear range of response, and/or capability of measuring at physiological pH. Regarding biosensing platforms, the main studies have focused on electrochemical approaches as an alternative to liquid chromatography–tandem mass spectroscopy and fluorometric techniques, which are the gold standard in glycine monitoring [36]. Although these techniques present the best detection limits (see Table 1), they still involve the disadvantages of highly expensive equipment, time-consuming analysis, and highly trained operators. Voltammetry/amperometry and metal-based redox mediators are predominantly preferred for electrochemical sensors. However, most of them still lack appropriate selectivity, showing interferences in analysing biological samples. Thus, the biosensor’s sensitivity to Gly could be enhanced by using a different enzyme with a higher affinity since different enzymes exhibit varying affinities to the same substrate. Some reports established the enhancement of selectivity using enzymes as elements of recognition, such as glycine oxidase. However, this enzyme is active for glycine, d-alanine and d-proline, sarcosine, and N-ethylglycine. In terms of stability, glycine oxidase can be stored at 4 °C for short periods, hindering [43]. Additionally, sensitivity to the analyte could be increased by utilizing antibodies. Research has shown that antibody-based SPR biosensors possess higher sensitivity to other analytes in blood plasma. For instance, Sankiewicz et al. [44] achieved a LOD of 0.07 ng mL^−1^ and LOQ of 0.23 ng mL^−1^ using rabbit anti-leptin antibody immobilized on a gold chip via a cysteamine linker, employing the same cross-linkers as in our work.

In recent years, there has been a growing demand for new point-of-care devices that deliver real-time information. Despite this, most clinical data are still analysed using laboratory equipment, highlighting the need for alternative strategies and new (bio)sensing recognition elements. Our study not only presents the surface plasmon resonance (SPR) platform as an advancement in methods for glycine detection but also suggests the laccase enzyme as a receptor for glycine detection for the first time. Although our biosensor could not achieve the limit of detection of glycine within the physiological range, the system may also act as an alarm for monitoring cases where glycine levels exceed the normal range. Also, it could be applicable in industries such as supplements or food testing, particularly in whey samples [51]. Whey powder contains high glycine levels, as Liu et al. [52] reported at a concentration of 2.3% *w*/*w*. Further perspectives suggest that different enzymes, such as glycine oxidase (GlyOx), could be tested using the optical approach that utilizes an SPR platform. This enzyme has been reported for glycine detection using an electrochemical platform that generated amperometric responses [48].

## 4. Conclusions

A novel enzyme SPR-based biosensor was developed in this study to detect and quantify glycine in human blood serum. The biosensor simplifies detection and uses minimal sample pretreatment and only a few reagents. It has a limit of detection (LOD) of 9.95 mM and a limit of quantification (LOQ) of 33.19 mM, with a working range of 0 to 500 mM, demonstrating a strong linear correlation (R^2^ = 0.9766) in the calibration curve. Validation using a spiked blood serum sample resulted in 97.64 ± 7.71% recovery percentage. The biosensor’s signal intensity remained consistent for 21 days after 60 uses, indicating its reusability for analysing multiple samples. While the current LOD and LOQ may not meet the medical standards of healthy patients, it could act as an alarm in cases where glycine-related disease causes high levels in the blood. The biosensor also shows promise in analysing food samples characterized by high glycine concentrations, such as whey, processed food, and supplements. Consequently, future work must optimize the protocol testing of different conditions (e.g., pH, use of mediators, different bioreceptors) to establish strategies to minimize detrimental factors.

## Figures and Tables

**Table 1:** Analytical parameters comparison between different methods reported for the determination of glycine.

Methods	LOD (mM)	Dynamic Range (mM)	Reference
Laccase-based surface plasmon resonance	9.953	0–500	This study
Attenuated total reflection (ATR) spectroscopy	3.463	0–666	[45]
Near-infrared (NIR) spectroscopy	2.930	0–666	[45]
Amperometry with electrode of Ni(OH)2	0.030	0.10–1.2	[46]
Differential pulse voltammetry with glassy carbon electrode-modified Co(OH)O nanoparticles	0.010	0.020–1.5	[47]
Amperometric sensor with carbon electrode modified with glycine oxidase	0.011	0.020–0.50	[48]
Cyclic voltammetry with carbon electrode modified with Prussian Blue -Chitosan–lecithin nanocomposite	0.008	0.007–0.240	[49]
Liquid chromatography (LC)–quadrupole-time-of-flight–tandem mass spectrometry (MS/MS)	0.009	0.033–0.666	[50]

## Data Availability

The data presented in this study are available upon request from the corresponding author.
